# Factors associated with lung cytology as obtained by non-endoscopic broncho-alveolar lavage in group-housed calves

**DOI:** 10.1186/s12917-019-1921-x

**Published:** 2019-05-24

**Authors:** Katharina van Leenen, Laura Van Driessche, Lieze De Cremer, Linde Gille, Christien Masmeijer, Filip Boyen, Piet Deprez, Bart Pardon

**Affiliations:** 1 Department of Large Animal Internal Medicine, Faculty of Veterinary Medicine, Ghent University, Salisburylaan 133, 9820 Merelbeke, Belgium; 2 Department of Pathology, Bacteriology and Avian Diseases, Faculty of Veterinary Medicine, Ghent University, Salisburylaan 133, 9820 Merelbeke, Belgium

**Keywords:** Neutrophilia, Respiratory disease, Total nucleated cell count, Eosinophils, Thoracic ultrasound, *Pasteurella multocida*

## Abstract

**Background:**

Respiratory infections are the main indication for antimicrobial use in calves. As in humans and horses, studying inflammation of the deep airways by lung cytology raises the possibility of preventing respiratory disease and targeting its treatment in the future. Whether lung cytology findings coincide with clinical signs and lung ultrasonographic findings is currently unknown. Therefore, the objective of the present study was to determine the association of lung cytology with clinical signs, lung consolidation and broncho-alveolar lavage fluid (BALf) characteristics (including bacteriology).

A total of 352 indoor group-housed calves aged between 1 and 6 months from 62 conveniently selected commercial herds were included in this cross-sectional study. Clinical examination, thoracic ultrasound and bacteriology and cytology on non-endoscopic broncho-alveolar lavage (nBAL) samples were performed.

**Results:**

Pneumonia, defined as presence of ultrasonographic lung consolidations ≥1 cm in depth, affected 42.4% of the calves. Mean BALf neutrophil percentage was 36.6% (SD 23.8; R 0–97.4) and only a positive induced tracheal cough reflex (*P* = 0.04), standing posture (*P* = 0.03) increased breathing rate (*P* = 0.02) and isolation of *Pasteurella multocida* (*P* = 0.005), were associated with increased neutrophil percentage. No significant associations between lung ultrasonographic findings and cytology results were present, except for presence of basophils in BALf and consolidation of > 3 cm in depth (OR = 2.6; CI = 1.2–5.6; *P* = 0.01). Abnormal lung sounds were associated with detection of eosinophils in BALf (OR = 2.8; CI = 1.0–8.1; *P* = 0.05). Total nucleated cell count (TNCC) (*P* <  0.001) was positively and macrophage percentage (*P* = 0.02) negatively associated with volume of lavage fluid recovered. Macroscopic blood staining of BALf increased TNCC (*P* = 0.002) and lymphocyte percentage (*P* = 0.001).

**Conclusions:**

Only a limited number of clinical signs and ultrasonographic findings were associated with nBAL cytology. BALf cytology offers additional and distinct information in calves aiding in detection and prevention of respiratory conditions. In this population, selected from herds not reporting any recent respiratory illness, a high number of calves had ultrasonographic lung consolidation and high neutrophil percentage in BALf, suggesting that subclinical disease presentations frequently occur.

**Electronic supplementary material:**

The online version of this article (10.1186/s12917-019-1921-x) contains supplementary material, which is available to authorized users.

## Background

Respiratory tract infections have severe economic consequences for the cattle industry worldwide [1–3]. In recent years, their importance has even increased because they are the main reason for antimicrobial use in calves and feedlot animals [4, 5]. Reduction of antimicrobial use in food producing animals is a top priority of the European Union [6]. Respiratory tract infections induce inflammation of the upper, and in many cases also the lower respiratory tract [7–9]. However, besides infections airway inflammation can be caused by non-infectious factors, such as air pollutants, related to bad stable environments [10–12]. Airway inflammation of various origin can reduce pulmonary function, induce mucus accumulation and reduce ciliary activity, potentially resulting in secondary (bacterial) pneumonia, as demonstrated in humans and horses [10, 13]. In cattle, studying airway inflammation is largely unexplored territory. Notwithstanding, knowledge on airway inflammation has the potential to help the industry towards a more preventive approach and to better targeted antimicrobial treatment. The reference method to study inflammation of the deep airways, is cytology on broncho-alveolar lavage (BAL) samples [14–17]. This can be done by bronchoscopic methods or by the use of non-endoscopic techniques, using a BAL-catheter [18, 19]. Both BAL technique and flushing volume greatly influence cytological results [17, 20]. For calves, available publications on lung cytology only deal with bronchoscopic BAL techniques, in a limited number of animals [8, 21]. However, in several Western European countries, veterinarians increasingly use a non-endoscopic BAL method (nBAL). Sterilisable custom-made or commercially available BAL catheters are used, allowing sampling of multiple animals in a limited time frame at low cost [22]. This blind technique does not systematically sample the diaphragmatic lung lobes, but a random lung lobe [23]. Today, practitioners use this sampling technique to identify pathogens involved in outbreaks of respiratory disease, despite concern on possible contamination of nBAL samples by nasal passage [24]. Nevertheless, in contrast to bronchoscopic procedures, nBAL has found its way to the field, opening possibilities to include lung cytology as an additional parameter for the management of respiratory diseases in calves. For welfare reasons, veterinarians prefer a small flushing volume (30 mL) for nBAL [22]. This type of mini-BAL has recently also gained access in human medicine for both lung cytology as bacteriology for example in the management of ventilator associated pneumonia or in immunocompromised patients [25–27]. To what extent lung cytology coincides with clinical signs or the more recently introduced on-farm lung ultrasonographic findings [28, 29], is currently unexplored. Whether nBAL cytology and bacteriology results are associated is also unknown, in animals as in humans.

The objective of the present study was to determine whether clinical signs, lung ultrasound, and broncho-alveolar lavage fluid (BALf) characteristics (including bacteriology) are associated with lung cytology of nBAL samples in a population of indoor group-housed calves.

## Methods

### Sample size calculation, study design, and animals

The sample size required to detect a 10% difference in neutrophil percentage between calves with and without a given risk factor, using 5% as a reference as seen in healthy horses [16], was 139 animals in each test group (with 95% confidence and 80% power). Sample size was further increased to 352 animals, to assure inclusion of the most common bacterial pathogens in the sample and the possibility to explore factors with multiple categories. A cross-sectional field study was performed on 62 conveniently selected commercial herds (23 dairy, 23 beef, 14 mixed and 2 veal) between January and April 2017. Herds were conveniently selected, with the help of different local veterinary practices, on willingness to cooperate and covered mainly the provinces West and East Flanders (Belgium). The only inclusion criteria was absence of an epidemic episode of respiratory disease in the last 2 months to avoid massive neutrophilia in the majority of the samples. An epidemic episode of respiratory disease has been defined as 20% new cases of respiratory disease in the same stable or age category in a 24-h period. Animal selection criteria were indoor group-housing, absence of oral or systemic antimicrobial treatment in the past 2 weeks and age between 1 and 6 months. The objective was to sample 8–10 calves per farm, housed in the same pen or 2 adjacent pens. If less animals were present all calves were sampled, if more than 10 calves were present in one pen animals were selected randomly. Veal calves were group-housed on a slatted floor and fed milk replacer, concentrates and roughage according to European legislation (EC2008–119). Beef and dairy calves were both group-housed on straw bedding and fed milk replacer, concentrates and roughage with substantial variation between farms, all calves had ad libitum access to water. All sampling techniques and the study protocol were revised by the Ethical Committee of the Faculty of Veterinary Medicine, Ghent University and permitted under experimental licence number EC2016–89. After the study all calves remained on the farm, no animals were sacrificed for this study.

### Clinical examination and thoracic ultrasonography

For each calf data on the following clinical signs was collected: mentation (alert versus depressed; depressed = decreased activity, eye closure, reduced awareness of the environment, lowering of the head), posture (standing; sternal recumbency; lateral recumbency), head tilt (present; absent), position of the ears (normal; unilaterally drooped or bilaterally drooped), nasal discharge (absent; unilateral; bilateral), ocular discharge (absent; unilateral; bilateral), type of ocular or nasal discharge (serous; seromucous; mucopurulent; purulent), spontaneous cough (present; absent), breathing frequency (in breaths per minute), heart rate (in beats per minute), rectal temperature (°C), induced laryngeal cough reflex (positive; negative), induced tracheal cough reflex (positive; negative), faecal consistency (normal; pasty; watery diarrhea) and lung auscultation (normal versus abnormal; abnormal = increased respiratory sounds, pleural friction sound or presence of wheezes or crackles). A positive induced laryngeal or tracheal cough reflex was defined as a single induced cough following manual compression of the larynx or trachea, respectively.

Thoracic ultrasound was performed with a linear probe with a frequency of 7.5-MHz (Tringa Linear Vet®, Esaote, the Netherlands), set at 8 cm of depth, using isopropyl alcohol (70%) as a transducing agent as previously described [30]. Presence of lung lesions was documented according to location (dorsal;ventral / left;right) and size of the lesion. Size was categorized using an adapted 6-point scale ultrasonographic lesion score (ULS). The definitions used for the ultrasonographic lesion score were as proposed by Ollivett et al., [21] with slight modifications: normal (ULS 0): only normal reverberation artefacts; comet tails (ULS 1): < 8 comet-tail artefacts in one field without presence of hypoechoic consolidations; interstitial syndrome (ULS 2): diffuse (> 8) comet-tail artefacts in one field without presence of hypoechoic consolidations; small consolidation (ULS 3): hypoechoic consolidation < 1 cm in depth; moderate consolidation (ULS 4): hypoechoic consolidation 1–3 cm in depth; severe consolidation (ULS 5): hypoechoic consolidation > 3 cm in depth. Consolidation depth was measured in a dorso-ventral plane using the grid on the screen of the ultrasound. Pleural effusion, characterized as a line of hypoechoic fluid between the lung and the pleural interface, was described as being absent or present.

### Broncho-alveolar lavage technique and BALf analysis

BALf was collected by a non-endoscopic technique using a reusable custom made polytetrafluorethylene catheter of 1.5 m length with a 12G- catheter stylet and an inner and outer diameter of 2 mm and 4 mm, respectively (VWR, Leuven, Belgium). The procedure was performed in standing, unsedated animals by the same veterinarian, using a new sterilized catheter for each calf as described previously [23]. Briefly, after the nostril was cleaned with 70% isopropyl alcohol the catheter was inserted into the ventral meatus of the nose and advanced to the bronchi until the wedge position was reached. One aliquot isotonic sterile saline was instilled using a volume of 0.6 ml/kg body weight. After instillation the fluid was immediately aspirated and if no fluid was recovered, another 20 mL of saline was instilled. The volume of instilled and recovered BALf and macroscopically visible blood staining of the sample were recorded for each animal. If the esophagus was accidently entered during the attempt to access the trachea a new sterilized catheter was used and the sampling process repeated from the start to avoid contaminated samples. Samples were transported in plastic tubes on ice and processed within 12 h after sampling.

Total nucleated cell count (TNCC) of the recovered lavage fluid was determined manually using a haemocytometer. The sample was vortexed and 1 μL of BALf was diluted with 10 μL Türk’s solution (Merck KGaA, Darmstadt, Germany) and counted manually using a Bürker counting chamber (Marienfeld GmbH & Co. KG, Lauda-Königshofen, Germany). Diff-Quick (Merck KGaA, Darmstadt, Germany) stained cytocentrifuge (Shandon Scientific, London, UK) preparations of BALf (1200 rpm for 10 min) were made and a total of 400 nucleated cells was counted at × 100 magnification to calculate the differential cell count [18]. All specimens were counted by the same observer (trained veterinarian).

Bacterial culture was performed using Columbia blood agar enriched with 5% sheep blood (Oxoid™, Hampshire, UK) for isolation of *Pasteurellaceae* and pleuropneumonia-like organism agar (PPLO) (Difco™, Becton Dickinson and Company, Franklin Lakes, NJ, USA), for isolation of *Mycoplasmataceae.* Incubation was done at 35 °C and 5% CO_2_, overnight and for 5 days, for *Pasteurellaceae* and *Mycoplasmataceae,* respectively. *Mycoplasma bovis* was identified by inoculating colonies on a PPLO agar enriched with polysorbate 80 (Difco™, Becton Dickinson and Company, Franklin Lakes, NJ, USA), followed by microscopic identification of growing colonies by their typical morphology [31]. Species confirmation of *Pasteurellaceae* was performed using Matrix-Assisted Laser Desorption Ionization-Time of Flight Mass Spectrometry (MALDI-TOF MS) (Brüker Daltonik GmbH, Bremen, Germany). Bacterial cultures for *Pasteurellaceae* were interpreted as negative, polymicrobial and dominant or pure cultures of *Pasteurella multocida, Mannheimia haemolytica* and *Histophilus somni* as described previously [22]. *M. bovis* cultures were interpreted as positive or negative.

### Statistical analysis

All data were entered in a spreadsheet (Excel, Microsoft Inc. Washington, USA) and transferred to SAS 9.4 (SAS Institute Inc., Cary, N.C, USA) for statistical analysis. To determine the relationship between TNCC and the different cell populations (in %) scatter plots were made and linear regression applied (PROC REG). Outcome variables were checked for a normal distribution and log + 1 transformed when required. Samples were considered positive for eosinophils if > 1% eosinophils were present in BALf and were considered positive for basophils if any of these cells were present in the 400 cells counted [32, 33].

Five multivariable linear regression models (PROC MIXED) were made with TNCC, neutrophil percentage, macrophage percentage, lymphocyte percentage, and percentage of epithelial cells as outcome variables. Predictors were clinical signs, BALf characteristics and ultrasonographic parameters. Thirteen clinical signs were tested: mentation (alert; depressed), posture (standing; sternal recumbency), head tilt (present; absent), ear position (normal; unilateral droopy ears; bilateral droopy ears), nasal discharge (absent; unilateral; bilateral), ocular discharge (absent; unilateral; bilateral), spontaneous cough (present; absent), breathing frequency, heart rate, temperature, induced tracheal cough reflex (negative; positive), induced laryngeal cough reflex negative; positive) and lung auscultation (normal; abnormal). Six BALf characteristics were tested: volume of BALf recovered (in mL), blood staining (absent; present), *P. multocida* isolation (negative; positive), *M. haemolytica* isolation (negative; positive), *H. somni* isolation (negative; positive), and *M. bovis* isolation (negative; positive). Four different binary outcomes were created based on ultrasonographic findings: ultrasonographically normal lung (reverberation artefacts and <  8 comet-tail artefacts in one image), diffuse comet-tail artefacts (> 8 in one image), lung consolidation (if a consolidation was present the depth in centimetres was noted), pleural effusion (absent; present). In each model herd was added as a random effect to account for clustering of calves within a herd. In a first step the association of the different predictors with the outcome variable was tested univariably. Continuous parameters (temperature, breathing rate, heart rate and BALf volume) were tested both continuously and categorically based on quartiles and receiver operating characteristics curve with the Youden’s index to determine optimal cut-off to create a binary variable. All parameters with *P* <  0.20 were withheld for the next step. Pearsons and Spearman correlation were determined, and of predictors correlated over 0.6, only the most significant one was withheld for the multivariable model. The multivariable model was built stepwise backwards, gradually excluding not significant variables. Significance was set at *P* <  0.05 and 0.05 < *P* <  0.10 was considered a trend. Pairwise comparisons between different categories of significant effects were made using Bonferonni corrections. Biologically plausible interactions between significant main effects were tested. Model fit was assessed by visual inspection of residual plots and normality testing of residuals.

For BALf eosinophils and basophils a multivariable logistic regression was used. Samples containing > 1% eosinophils were considered positive (increased), as in humans and horses [32, 33]. For basophils, a sample was considered positive if any of these cells were seen in the 400 cells counted [33]. A generalized linear mixed model (PROC GLIMMIX) was used with binomial distribution and logit link function with Wald’s statistics for type 3 contrasts. Herd was added as a random factor to account for clustering. First, the same predictors as mentioned above were tested in univariable analysis. The same selection and significance criteria as mentioned above were used for the further model building procedure. Model fit was evaluated using the Hosmer-Lemeshow goodness-of-fit test for logistic models. All possible and biologically relevant interactions between significant main effects were tested.

## Results

### Animals and herds

On 62 farms a total of 352 animals were sampled, consisting of 50.1% (178/352) Holstein-Friesian calves, 44.3% (156/352) Belgian Blue calves and 5.1% (18/352) mixed breed calves. Of the Holstein-Friesian calves 5% (9/178) originated from a veal calf facility, the remaining 95% (169/178) from a dairy farm. On 87.1% (54/62) of the farms less than eight calves were present in one pen or in adjacent pens. Therefore all eligible calves were sampled on these farms. None of the farms had ten or more calves in one pen or adjacent pens. Of the calves, 4.3% (15/352) were aged 4 weeks or less, 35.8% (126/352) were aged between 4 and 8 weeks and 59.9% (211/352) of the calves were more than 8 weeks old.

### Clinical signs and ultrasonographic lesions

**Fig. 1:**
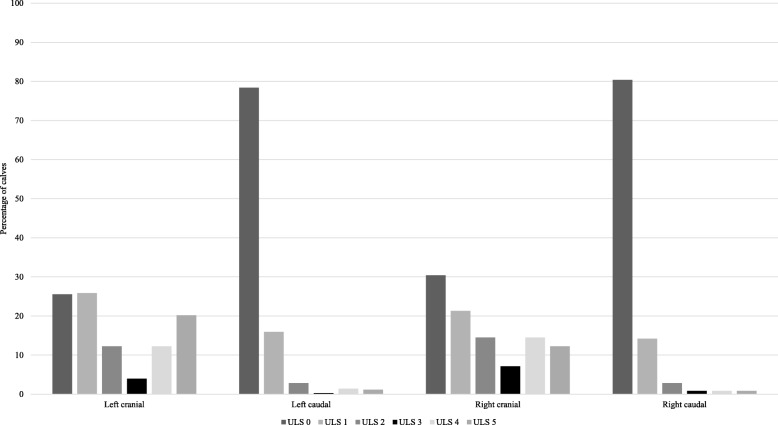
Anatomical distribution of lung lesions grouped by ultrasound lesion score (ULS) from 352 indoor group-housed calves. ULS 0 = only normal reverberation artefacts; ULS 1 = < 8 comet-tail artefacts in one image present; ULS 2 = diffuse (> 8) comet-tail artefacts without presence of hypoechogenic consolidations; ULS 3 = hypoechogenic consolidation < 1 cm in depth; ULS 4 = hypoechogenic consolidation 1–3 cm in depth; ULS 5 = hypoechogenic consolidation > 3 cm in depth

Mean rectal temperature was 39.0 °C (standard deviation [SD] = 0.5; range [R] = 36.6–41.2) and mean breathing frequency 35 breaths/minute (SD, 24; R, 14–116). In 51.7% (182/352) of the animals at least one of the recorded clinical signs was present. Uni- or bilateral nasal discharge was the most frequently observed sign, present in 22.2% (78/352) of the calves, followed by spontaneous cough in 21.0% (74/352), uni- or bilateral drooped ears in 5.7% (20/352) and depression in 4.2% (15/352). A positive tracheal reflex could be induced in 14.2% (50/352) of the animals whereas the laryngeal reflex was positive in only 2.6% (9/352). Thoracic ultrasound demonstrated lung consolidation with a depth of ≥1 cm (ULS 4–5) in 42.4% (149/352) and a consolidation > 3 cm (ULS 5) in 23.9% (84/352) of the calves. Pleural effusion was seen in 4.5% (16/352) of the calves. The anatomical distribution of the ultrasonographic lesions is presented in Fig. 1. No significant age, breed or herd effects on the presence of lung consolidations (≥1 cm in depth) could be demonstrated. For lung consolidations > 3 cm in depth, significant breed (dairy > beef breeds (odds ratio (OR) = 5.6 (95% confidence interval (CI) = 2.0–16.2; *P* <  0.001) and herd effects (*P* <  0.001) could be demonstrated, but not age effects (*P* = 0.14).

### BALf characteristics, cell counts and bacteriology

The average percentage of instilled fluid was 38.8 ml (SD, 5.2 ml; R, 25–80 ml) and in 1.7% (6/352) of the calves an additional 20 ml of saline needed to be instilled due to insufficient recovery. Mean percentage of instilled fluid recovered was 33.5% (SD, 9.9; R, 12.3–73.8) and 11.1% (39/352) of the samples were macroscopically blood stained. Blood stained samples were significantly more frequent in calves aged > 8 weeks (14.7%; [31/211]) compared to calves aged between 4 and 8 weeks (5.6%; [7/126]) (*P* = 0.04).

Mean TNCC was 1.9 × 10^9^ cells/L (SD, 1.7; R, 0–13.7). The ULS score was not associated with TNCC. Mean BALf differential cell percentage was 42.8% macrophages (SD, 18.9; R, 2.4–92.3), 36.6% neutrophils (SD, 23.8; R, 0–97.4), 5.4% lymphocytes (SD, 5.2; R, 0–45.8), 0.3% eosinophils (SD, 0.8; R, 0–9.1), 0% basophils (SD, 0.1; R, 0–0.7) and 14.9% epithelial cells (SD, 13.0; R, 0–95.9), as shown in Additional file 1: Table S1. Of the calves 5.7% (20/352) showed eosinophilia (> 1% eosinophils) and 10.5% (37/352) had basophils in their BAL sample. Calves with eosinophils and basophils in their BALf were present on 25.8% (16/62) and 35.5% (22/62) of the farms, respectively.

**Fig. 2:**
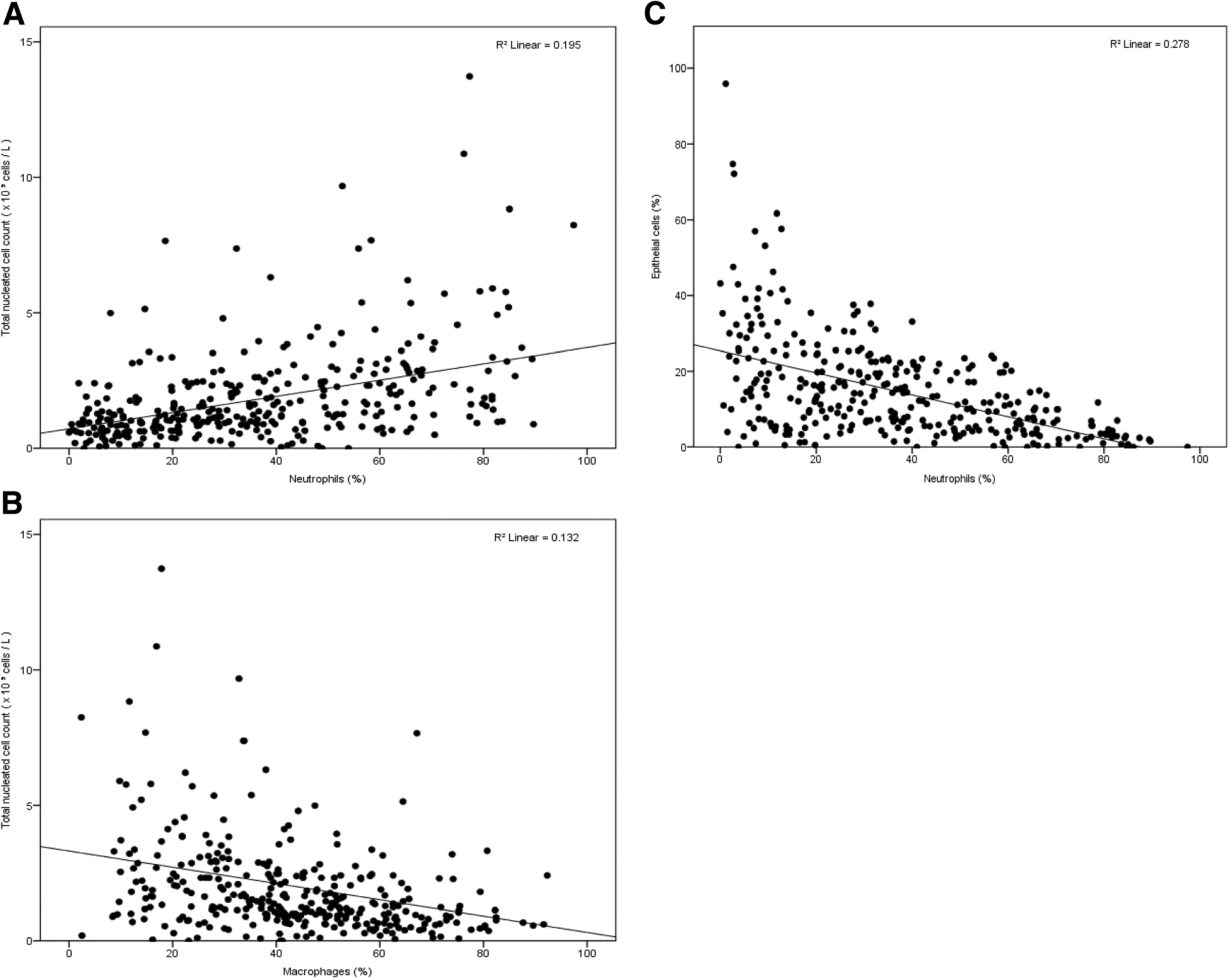
Linear associations between different broncho-alveolar lavage fluid cellular components from 352 indoor group-housed calves. Significant associations were present for total nucleated cell count and neutrophil percentage (**a**), total nucleated cell count and macrophage percentage (**b**) and epithelial cell percentage and neutrophil percentage (**c**). No significant associations were found between other cell types

A positive correlation was found between neutrophil percentage and TNCC (r = 0.20) whereas macrophage percentage was negatively correlated with TNCC (r = 0.13). Between the percentage of epithelial cells and percentage of neutrophils in BALf a negative correlation was noted (r = 0.28) (Fig. 2). No other significant correlations between the different cell types were present. No significant differences were found between breed, age, clinical signs and BALf cellular characteristics, as shown in Additional file 2: Table S2. No significant differences were found between BALf differential cell count of lungs without ultrasonographic consolidations versus lungs which did show these ultrasonographic lesions or pleural effusion.

A pure or a dominant culture for *Pasteurellacea* were present in 16.8% (59/352) and 33.5% (118/352) of the BALf samples, respectively. In 40.6% (143/352) of the samples a polymicrobial result was obtained and 9.0% (32/352) of the BALf samples were bacteriologically negative. *P. multocida* was isolated from 31.2% (110/352) of the samples, *M. haemolytica* from 14.2% (50/352), *H. somni* from 2.3% (8/352) and 3.1% (11/352) of the samples were positive for *M. bovis.* Mixed infections were present in 5.1% (18/352) of the samples and consisted of *M. bovis* and *P. multocida* (1.4% [5/352]), *P. multocida* and *M. haemolytica* (1.4% [5/352]), *M. bovis* and *M. haemolytica* (1.1% [4/352]), *M. haemolytica* and *H. somni* (0.6% [2/352]) and *P. multocida* and *H. somni* (0.6% [2/352]) combinations. Pathogen isolation rates were not linked with clinical signs or ultrasonographic findings.

### Factors associated with TNCC and differential cell counts

Log transformation was needed for TNCC, neutrophil percentage, lymphocyte percentage and epithelial cell percentage. Macrophage percentage was normally distributed. For eosinophil and basophil percentage no transformation to a normal distribution was possible, hence it was opted to analyse them as binary outcomes. When performing the final mixed model analysis 17 calves were excluded for the analysis of BALf neutrophils, lymphocytes and epithelial cells and 20 calves were excluded for the analysis of BALf TNCC and macrophages, due to incomplete information on some of the predictors studied, resulting in 335 and 332 calves for final analyses, respectively.

The final multivariable models for TNCC (Table 1), neutrophil percentage (Table 2), macrophage percentage (Table 3), lymphocyte percentage (Table 4), epithelial cell percentage (Table 5), presence of > 1% eosinophils and presence of basophils (Table 6) are available in the respective tables. Older calves (> 8 weeks) had a significantly higher lymphocyte percentage, but no age effects on other cell types were noticed (Table 4). For BALf characteristics, the higher the recovered BALf volume, the higher TNCC and the lower the macrophage percentage were (Tables 1 and 3). Macroscopic blood staining of BALf, significantly increased TNCC and lymphocyte percentage and was associated with the presence of basophils (Tables 1, 4 and 6). Of all clinical symptoms recorded only a positive induced tracheal cough reflex, standing position of the calf and increased breathing rate remained associated with cellular changes in BALf in the multivariable models. A positive induced tracheal reflex was associated with an increased TNCC and neutrophil percentage and decreased epithelial cell percentage (Tables 1, 2 and 5). An increased breathing frequency was associated with a decreased percentage of epithelial cells and an increased neutrophil percentage (Tables 2 and 5). Clinical signs frequently used to identify calves suffering from respiratory disease such as nasal and ocular discharge, fever and spontaneous cough were not associated with any of the studied outcomes. Of the upper quartile of calves with the highest BALf neutrophil percentage 21.6% (19/88) had an increased breathing frequency (> 44 bpm) and 19.3% (17/88) a positive induced tracheal reflex.

**Table 1:** Final multivariable linear model for the association of clinical signs and broncho-alveolar lavage fluid characteristics with total nucleated cell count (cells × 10^9^ L) in broncho-alveolar lavage fluid of 332 group-housed calves

Variable	Category	Regression coefficient β (SE)	*P*-value	LSM estimate (SE)
Intercept		1.6 (0.14)	< 0.001	
Tracheal cough reflex	Negative	Referent		1.7 (0.04)
	Positive	0.24 (0.07)	0.003	2.3 (0.08)
BALf blood staining	Absent	Referent		1.7 (0.04)
	Present	0.26 (0.08)	0.002	2.4 (0.08)
Volume lavage fluid recovered (/mL)		0.03 (0.01)	< 0.001	-

*BALf* broncho-alveolar lavage fluid. Random herd effect was not significant (*P* = 0.12)

**Table 2:** Final multivariable linear model for the association of clinical signs and broncho-alveolar lavage fluid characteristics with neutrophil percentage in broncho-alveolar lavage fluid of 335 group-housed calves

Variable	Category	Regression coefficient β (SE)	*P*-value	LSM estimate (SE)
Intercept		20.4 (0.14)	< 0.001	
*P. multocida* culture	Negative	Referent		22.7 (0.11)
	Positive	0.32 (0.10)	0.005	30.3 (0.13)
Tracheal cough reflex	Negative	Referent		22.7 (0.10)
	Positive	0.32 (0.15)	0.04	30.3 (0.16)
Posture	Sternal recumbency	Referent		21.4 (0.19)
	Standing	0.47 (0.19)	0.03	32.1 (0.08)
Breathing frequency (breaths/min)		0.01 (0.0)	0.02	-

Random herd effect was significant (*P <* 0.001)

**Table 3:** Final multivariable linear model for the association of clinical signs and broncho-alveolar lavage fluid characteristics with macrophage percentage in broncho-alveolar lavage fluid of 332 group-housed calves

Variable	Category	Regression coefficient β (SE)	*P*-value	LSM estimate (SE)
Intercept		57.7 (5.4)	< 0.001	
*P. multocida* culture	Negative	Referent		49.2 (2.1)
	Positive	- 4.5 (2.2)	0.04	44.7 (2.5)
Posture	Sternal recumbency	Referent		52.1 (3.7)
	Standing	- 10.2 (3.8)	0.007	41.8 (1.3)
Volume lavage fluid recovered (/mL)		- 0.6 (0.27)	0.02	-

Random herd effect was significant (*P <* 0.001)

**Table 4:** Final multivariable linear model for the association of clinical signs and broncho-alveolar lavage fluid characteristics with lymphocyte percentage in broncho-alveolar lavage fluid of 335 group-housed calves

Variable	Category	Regression coefficient β (SE)	*P*-value	LSM estimate (SE)
Intercept		6.2 (0.34)	< 0.001	
Mentation	Alert	Referent		4.1 (0.10)
	Depressed	- 0.41 (0.19)	0.05	2.6 (0.20)
Age	> 8 weeks	Referent		4.2 (0.12)
	4–8 weeks	- 0.29 (0.11)	0.02	3.1 (0.03)
	4 weeks	- 0.41 (0.27)	0.15	2.7 (0.27)
BALf bloodstaining	Absent	Referent		2.8 (0.12)
	Present	0.32 (0.12)	0.01	3.9 (0.16)

*BALf* broncho-alveolar lavage fluid. Random herd effect was significant (*P <* 0.001)

**Table 5:** Final multivariable linear model for the association of clinical signs and broncho-alveolar lavage fluid characteristics with epithelial cell percentage in broncho-alveolar lavage fluid of 335 group-housed calves

Variable	Category	Regression coefficient β (SE)	*P*-value	LSM estimate (SE)
Intercept		9.9 (0.25)	< 0.001	
Breathing frequency (breaths/min)		- 0.01 (0.0)	0.03	-
Tracheal cough reflex	Negative	Referent		10.9 (0.07)
	Positive	- 0.53 (0.16)	0.004	6.3 (0.15)

Random herd effect was significant (*P <* 0.001)

**Table 6:** Final multivariable logistic regression model for the association of clinical signs and broncho-alveolar lavage fluid characteristics with eosinophil and basophil percentage in broncho-alveolar lavage fluid of 352 group-housed calves

Cell type	Variable	Category	Calves (n =)	Percentage	OR	95% CI	*P*-value
Eosinophils ^a^	Lung auscultation	Normal	159	3.8%	Referent		
		Abnormal	193	8.8%	2.8	1–8.1	0.05
Basophils ^b^	*H. somni* culture	Negative	340	9.1%	Referent		
		Positive	12	50.0%	1.4	2.9–45.5	< 0.001
	BALf bloodstaining	Absent	313	9.3%	Referent		
		Present	39	20.5%	3.2	1.2–8.3	0.02
	Lung consolidation > 3 cm in depth	Absent	245	7.3%	Referent		
		Present	107	17.8%	2.6	1.2–5.6	0.01

*BALf* broncho-alveolar lavage fluid. Random herd effect was significant for eosinophils (*P* = 0.03) and not significant for basophils (*P* = 0.20)

^a^ Cut-off > 1% eosinophils

^b^ Cut-off = ≥ 1 basophil counted on 400 cells

*OR* odds ratio, *CI* confidence interval

**Fig. 3:**
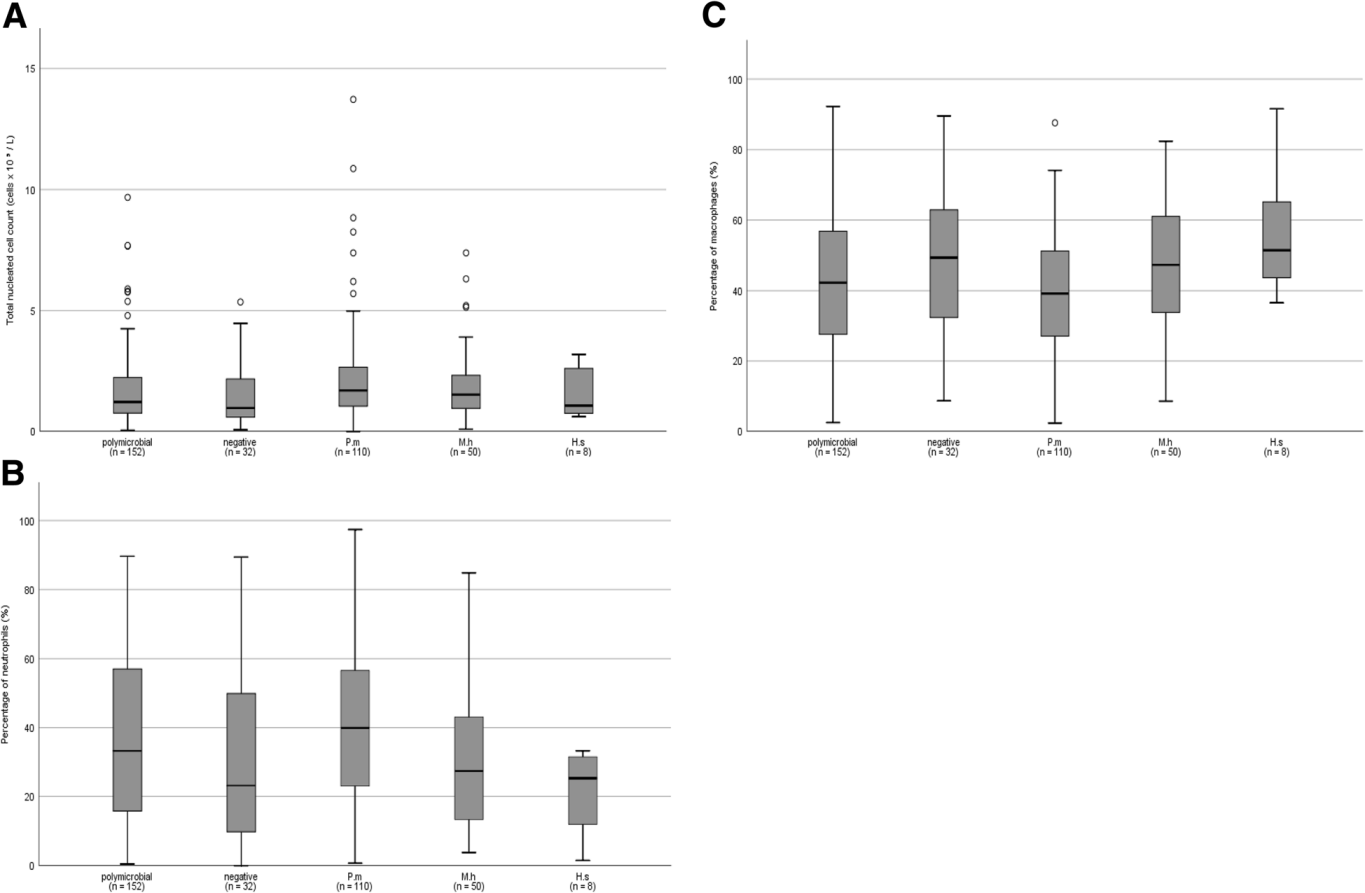
Total nucleated cell count and differential cell counts in broncho-alveolar lavage fluid from 352 indoor group-housed calves by isolated pathogen. **a** = total nucleated cell count, **b** = neutrophils, **c** = macrophages. Negative = no major pathogens could be isolated from the broncho-alveolar fluid; P.m = *Pasteurella multocida* isolation*;* M.h = *Mannheimia haemolytica* isolation; H.s = *Histophilus somni* isolation

For pathogen isolation (Fig. 3), only a positive *P. multocida* culture was associated with increased BALf neutrophil percentage and a reduction in macrophage percentage (Tables 2 and 3). *H. somni* isolation was associated with detection of basophils (Table 6). When testing the ULS as a categorical factor, no associations with any of the outcomes studied was found. When using different cut-offs to categorize the depth of ultrasonographic lung consolidation (> 1 cm; > 3 cm; > 6 cm), only consolidations with a depth of > 1 cm were univariably associated (*P* = 0.04) with an increased BALf neutrophil percentage. Only for the presence of basophils a significant association with lung consolidation > 3 cm (*P* = 0.001) remained in the multivariable model (Table 6).

## Discussion

In this study the association of clinical signs and lung ultrasonography with BALf cytology was explored, to gain insights in the potential added value of cytology for respiratory disease diagnostics and prevention in future work.

To include as much variation in the cytological variables as possible these associations were studied in a population of animals housed in herds with only endemic respiratory problems. Herds with endemic respiratory problems were defined as herds with no epidemic respiratory disease presentation in the last 2 months. Hence, the finding that in 42.4% of the calves lung consolidations (≥1 cm in depth) were present, despite that half of the calves studied did not show any abnormal clinical signs, was unexpected. This prevalence is higher than previously reported for 60-day-old Holstein heifers (20.2% (any consolidation) [34] and three-month-old Jersey heifers 27.6% (consolidation ≥1 cm) [1]. Notably, severe ultrasonographic lesions were more frequently present in Holstein-Friesian calves, whereas the Belgian blue breed has been known to be more susceptible for respiratory disease [35]. Possible explanations could be that dairy calves expressed clinical signs of pneumonia to a lesser extent, whereas beef farmers could have observed their calves closer and treated them more intense, given the greater value of the animals. Furthermore, the possibility of less awareness of (sub)clinical pneumonia in dairy calf farming in this region cannot be excluded. Besides breed, differences in management, housing and prophylactic regimens could have affected the prevalence of lung consolidation, nevertheless, this was outside the scope of this article.

This study showed that in this population of animals, housed in herds with only endemic respiratory problems, no association of lung ultrasonographic findings and lung cytology was found. This is in contrast to previous work on experimental animals using an endoscopic BAL technique [21]. Most likely these discordant findings are related to sampling of a random lung lobe with the nBAL technique, which could have resulted in sampling of a healthy lobe instead of a consolidated one in some animals. With the endoscopic lavage technique the consolidated lung lobe can be selectively sampled. An equally important finding was that not all of the commonly monitored respiratory signs were associated with inflammation (higher neutrophil percentage) of the lower airways. Only the induced tracheal cough reflex, posture of the calf and increased breathing rate were linked to neutrophil percentage. These signs might be more specific for lower respiratory tract inflammation, and potentially infection, which could stress their potential benefit for identification of animals requiring treatment, in contrast to signs like nasal discharge or fever. A tracheal cough reflex is triggered by stimulation of the irritated and inflamed mucosa of the lower airways, characterized by an influx of neutrophils. Calves that showed increased respiratory rates might have been suffering from hypoxia resulting from a more advanced pneumonia or acute interstitial pneumonia, likely of viral aetiology. Moreover, a standing position is often maintained in calves with breathing difficulties to facilitate breathing. However, in a substantial proportion of animals with high BALf neutrophil percentage these clinical signs were not demonstrated. A similar problem has been reported in horses with inflammatory airway disease, where BALf cytology is the only diagnostic means when no abnormal clinical signs are evident [16]. Off course, continuous monitoring of clinical signs might have improved the diagnostic accuracy of these signs to detect lung inflammation in our study. A limitation of this study was that selection bias (convenience sampling) and potentially also classification bias cannot be excluded, as all recordings, which are of subjective nature, were done by the same operator without blinding.

The absence of a reference framework for nBAL cytology was a serious limitation for our study. Given the absence of good criteria defining a calf with healthy airways, this issue applies to all sampling techniques and the cross-sectional design of this study appeared the best option to gain first insights. A clear finding was that BALf neutrophil levels exceeded values previously reported in calves [21, 36, 37]. BALf neutrophil percentage has been used as the primary indicator of inflammation and infection of the lower respiratory tract across species [7, 9, 37]. Suggested cut-off values defining BALf neutrophilia in calves are highly variable ranging from ≥4 to 39% [21, 38]. Neutrophil percentage might be influenced by the sampling volume and aliquot analysed. In human medicine, the first aliquot is often discarded since it might contain a higher bronchial component compared to subsequent aliquots [17, 39]. As the complete BAL sample was used and the first aliquot was not discarded in our study, this might have played a role. Furthermore, the bronchial component in BALf is increased when small volumes are used, as in this study, which could have resulted in an increased neutrophil percentage [17, 20]. However, the nBAL catheter consisted of a diameter of 4 mm suggesting it would be wedged in a smaller bronchus deep in the lung, where the bronchial component is relatively reduced compared to the volume of instilled fluid, diminishing the influence of these technical factors. Nasal passage might also have affected BALf neutrophil percentage, especially in animals with nasal discharge. We did not find any information in the literature regarding this aspect. In our study nasal discharge was not associated with increased BALf neutrophil percentage. Besides these technical aspects, both infection and inflammation due to non-infectious components of stable air, could explain the high neutrophil percentage in this population. A limitation of the study was that for financial reasons extensive viral examinations were not included, which might have explained a substantial proportion of the neutrophilia observed.

In addition to the aforementioned factors, bacterial infection would be a logical trigger of neutrophil influx, resulting in a very high neutrophil percentage [7–9]. The use of BAL in general, and nBAL specifically, for bacteriology has been controversial, mainly because of the potential risk of nasal contamination [24]. However, to the authors knowledge, no studies providing evidence or quantifying this risk of contamination are currently available. Mini-BAL procedures, comparable to the nBAL used in this study, have been used in human medicine and the interest in their potential use for microbiology purposes has been increased [40–42] In cattle the nBAL has already been used frequently for microbiology by practitioners, and gaining insights in its usefulness for this purpose, appears urgent. In our data, a significant association between *P. multocida* and an increased neutrophil and decreased macrophage percentage was demonstrated, as seen in another study [8]. Taking the limitations of nBAL towards nasal passage contamination into account, this might indicate that *P. multocida* could be a more virulent pathogen or a better secondary invader of airways that are already inflamed due to air pollution or viral infections, under the conditions of our study. In contrast to previous experimental studies, isolation of *M. haemolytica* was not linked to BALf neutrophil percentage, potentially due to differences in virulence between strains [43, 44]. *H. somni* isolation was not associated with neutrophilia, but increased both lymphocyte and basophil percentage. This might point towards a different type of immune response against *H. somni*. Given the low number of *H. somni* and *M. bovis* positive cases, the absence of an association between isolation of *H. somni* and *M. bovis* and an increasing neutrophil percentage should be interpreted with care.

Similar to BALf neutrophil percentage, TNCC counts exceeded values reported in previous work with the endoscopic technique [21]. Cut-off values for TNCC have also not been established for calves, and they depend on the technique used [24, 45]. Our results showed that unlike the differential counts, TNCC values were mainly influenced by technical factors. TNCC increased with increasing amount of recovered lavage volume and macroscopically visible blood staining. This blood staining was most likely caused by excessive vacuum (manual aspiration) or by the fragility of inflamed respiratory mucosa.

Eosinophil and basophil percentages in BALf from young calves were studied for the first time in this paper. Abnormal lung auscultation was associated with a BALf eosinophil percentage > 1%. Wheezes, as induced by bronchoconstriction, were heard in 25% (6/24) of these calves. Next to parasitic infections, these findings could point towards the existence of asthmatic syndromes in calves, as well-known in humans and horses [16, 46, 47]. Moreover, basophils have played a role in asthmatic syndromes and recent work from human medicine showed that basophils were activated in presence of suboptimal doses of allergens and bacteria [48]. Possibly, the observed association between *H. somni* isolation and presence of basophils in BALf points towards a similar mechanism in calves.

Finally, an age effect for BALf cytological composition has been observed, characterized by higher lymphocyte and lower macrophage levels in BALf of older calves. The same age effects were demonstrated in peripheral blood [49]. The possibility, that the observed age effect for lymphocytes was due to more frequent blood staining of BALf in calves older than 8 weeks, cannot be excluded in our study. In future studies this should be accounted for when aiming to set cytology reference values for a certain BAL technique.

## Conclusions

Cytology findings as determined by an nBAL method, were only associated to a limited extent with ultrasonographic findings and selected clinical signs (positive tracheal reflex, standing position and breathing rate). BALf cytology offers additional information to the analysis of respiratory problems in calves, potentially aiding in better prevention and targeted treatment in the future. In this population, selected from herds not reporting any recent respiratory diseases, a high prevalence of lung consolidation and animals with neutrophilia was detected, pointing towards issues with possible unawareness of the problem or subclinical disease presentations.

## Additional files

Additional file 1: **Table S1.** Broncho-alveolar lavage fluid total nucleated cell count and differential cell counts according to ultrasonographic lesion score (ULS) based on 352 group-housed calves. (DOCX 26 kb)

Additional file 2: **Table S2.** Descriptives of broncho-alveolar fluid cellular characteristics from 352 group-housed calves. (DOCX 37 kb)

## Abbreviations

BAL: Broncho-alveolar lavage
BALf: Broncho-alveolar lavage fluid
CI: Confidence interval
LSM: Least square means
nBAL: non-endoscopic broncho-alveolar lavage
OR: Odds ratio
PPLO: Pleuropneumonia-like organism
R: Range
SD: Standard deviation
SE: Standard error
TNCC: Total nucleated cell count
ULS: Ultrasonographic lesion score
