# Supplementary material for: Factors associated with lung cytology as obtained by non-endoscopic broncho-alveolar lavage in group-housed calves
Source: BMC Vet Res. 2019 May 24;15:167. doi: 10.1186/s12917-019-1921-x (PMC6534843; doi:10.1186/s12917-019-1921-x)
Supplement: Supplementary file 1 — Table S1. Broncho-alveolar lavage fluid total nucleated cell count and differential cell counts according to ultrasonographic lesion score (ULS) based on 352 group-housed calves. (DOCX 26 kb) [file 12917_2019_1921_MOESM1_ESM.docx]

| **Supplementary Table 1.** Broncho-alveolar lavage fluid total nucleated cell count and differential cell counts according to ultrasonographic lesion score (ULS) based on 352 group-housed calves. | | | | | | | |
| --- | --- | --- | --- | --- | --- | --- | --- |
| Variable | ULS | | | | | | |
|  | 0  (n = 60) | 1  (n = 72) | 2  (n = 44) | 3  (n = 27) | 4  (n = 65) | 5  (n = 84) | All calves  (n = 352) |

| Volume recovered (ml) | | 13.2 ± 4.1  (6.0 – 24.0) | 13.1 ± 3.5  (4.0 – 22.8) | 12.9 ± 3.1  (6.3 – 24.4) | 12.2 ± 4.5  (4.9 – 24.5) | 13.1 ± 4.2  (6.0 – 29.5) | 12.3 ± 3.4  (5.5 – 23.5) | 12.9 ± 3.8  (4.0 – 29.5) |
| --- | --- | --- | --- | --- | --- | --- | --- | --- |
| TNCC  (x10^9^ cells/L) | 1.7 ± 1.4  (0 – 6.2) | | 1.8 ± 1.7  (0 – 9.7) | 1.9 ± 1.7  (0.1 – 7.4) | 1.8 ± 1.5  (0 – 5.7) | 2.4 ± 2.1  (0 – 13.7) | 1.8 ± 1.7  (0.1 – 10.9) | 1.9 ± 1.7  (0 – 13.7) |
| Neu (%) | 39.3 ± 24.8  (1.9 – 84.4) | | 32.6 ± 23.2  (0 – 97.4) | 46.5 ± 28.2  (2.7 – 89.7) | 32.1± 19.3  (1.1 – 72.5) | 41.2 ± 25.2  (0.5 – 89.4) | 36.9 ± 20.9  (3.3 – 82.8) | 36.6 ± 23.8  (0 – 97.4) |
| Mac (%) | 39.8 ± 17.8  (12.0 – 75.3) | | 45.7 ± 20.0  (2.4 – 91.6) | 46.5 ± 22.5  (8.4 – 92.3) | 43.1 ± 6.8  (2.5 – 72.9) | 40.3 ± 19.5  (8.7 – 82.2) | 42.5 ± 16.5  (9.3 – 89.6) | 42.8 ± 18.9  (2.4 – 92.3) |
| Lym (%) | 6.1 ± 7.6  (0 – 45.8) | | 6.2 ± 5.3  (0.2 – 38.4) | 4.1 ± 4.5  (0 – 19.0) | 5.9 ± 4.9  (0.5 – 20.2) | 4.3 ± 4.4  (0 – 24.3) | 5.5 ± 3.5  (0 – 18.8) | 5.4 ± 5.2  (0 – 45.8) |
| Eo (%) | 0.3 ± 0.7  (0 – 4.4) | | 0.3 ± 0.9  (0 – 6.2) | 0.3 ± 0.6  (0 – 3.2) | 0.3 ± 0.6  (0 – 2.8) | 0.2 ± 0.3  (0 – 1.1) | 0.4 ± 1.2  (0 – 9.1) | 0.3 ± 0.8  (0 – 9.1) |
| Baso (%) | 0 ± 0.1  (0 – 0.6) | | 0 ± 0.1  (0 – 0.2) | 0 ± 0.1  (0 – 0.7) | 0.1 ± 0.1  (0 – 0.5) | 0.1 ± 0.1  (0 – 0.7) | 0 ± 0.1  (0 – 0.4) | 0 ± 0.1  (0 – 0.7) |
| Epi (%) | 14.5 ± 12.0  (0.6 – 53.1) | | 15.2 ± 13.8  (0 – 72.1) | 14.5 ± 11.6  (0 – 47.5) | 18.6 ± 19.5  (2.4 – 95.9) | 14.0 ± 13.6  (0 – 74.7) | 14.7 ± 10.7  (0 – 46.3) | 14.9 ± 13.0  (0 – 95.9) |
| Neu/Mac ratio | 1.0 | | 0.7 | 0.7 | 0.7 | 1.0 | 0.9 | 0.9 |

Values represent mean ± standard deviation (Min. – Max.). ULS 1 = < 8 comet-tail artefacts in one image present ULS 2 = diffuse (> 8) comet-tail artefacts without presence of hypoechogenic consolidations; ULS 3 = hypoechogenic consolidation < 1 cm in depth; ULS 4 = hypoechogenic consolidation 1 – 3 cm in depth; ULS 5 = hypoechogenic consolidation > 3 cm in depth. TNCC = total nucleated cell count; Neu = neutrophils;
Mac = macrophages, Lym = lymphocytes, Eo = eosinophils; Baso = basophils; Epi = epithelial cells.
