# Supplementary material for: Factors associated with lung cytology as obtained by non-endoscopic broncho-alveolar lavage in group-housed calves
Source: BMC Vet Res. 2019 May 24;15:167. doi: 10.1186/s12917-019-1921-x (PMC6534843; doi:10.1186/s12917-019-1921-x)
Supplement: Supplementary file 2 — Table S2. Descriptives of broncho-alveolar fluid cellular characteristics from 352 group-housed calves. (DOCX 37 kb) [file 12917_2019_1921_MOESM2_ESM.docx]

| **Supplementary Table 2.** Descriptives of broncho-alveolar fluid cellular characteristics from 352 group-housed calves. | | | | | | | | | | |
| --- | --- | --- | --- | --- | --- | --- | --- | --- | --- | --- |
| Parameter | Category | TNCC  (x10^9^ cells/L) | Neu  (%) | | Mac  (%) | | Lym  (%) | Eo  (%) | Baso  (%) | Epi  (%) |
| Breed | Holstein Friesian  Belgian Blue  Other | 1.8 ± 1.7  (0 –13.7)  2.0 ± 1.8  (0 – 9.7)  1.8 ± 1.0  (0.1 – 3.9) | 35.7 ± 22.5 (0 – 89.4)  37.6 ± 25.4  (0.5 – 97.4)  36.8 ± 23.5  (6.0 – 84.4) | 44.2 ± 18.3  (8.7 – 92.3)  41.2 ± 19.9  (2.4 – 91.6)  43 ± 14.6  (14.2 – 67.1) | | 6.5 ± 6.1  (0 – 45.8)  4.3 ± 3.8  (0 – 24.3)  3.9 ± 3.1  (0 – 9.9) | | 0.3 ± 0.8  (0 – 9.1)  0.3 ± 0.8  (0 – 6.2)  0.6 ± 1.1  (0 – 4.4) | 0 ± 0.1  (0 – 0.7)  0 ± 0.1  (0 – 0.7)  0 ± 0  (0 – 0) | 13.3 ± 10.9  (0 – 57.0)  16.6 ± 15.1  (0 – 95.9)  15.6 ± 10.9  (0.6 – 39.0) |
| Age | 4 weeks  4-8 weeks  >8 weeks | 1.5 ± 0.9  (0.4 – 3.3)  2.1 ± 2.0  (0.1 – 13.7)  1.8 ± 1.6  (0 – 9.7) | 26.3 ± 21.5  (0.7 – 80.9)  39.8 ± 25.9  (0.5 – 97.4)  35.9 ± 22.4  (0 – 86.1) | 55.6 ± 20.2  (13.3 – 87.6)  40.3 ± 19.6  (2.4 – 91.6)  43.1 ± 18.0  (9.7 – 92.3) | | 5.9 ± 11.6  (0 – 45.8)  4.2 ± 4.3  (0 – 38.4)  5.9 ± 4.6  (0 – 26.9) | | 0.1 ± .1  (0 – 0.2)  0.5 ± 1.2  (0 – 9.1)  0.2 ± .5  (0 – 4.4) | 0 ± 0.1  (0 – 0.2)  0 ± 0.1  (0 – 0.7)  0 ± 0.1  (0 – 0.7) | 12.1 ± 10.4  (0.9 – 35.8)  15.1 ± 14.8  (0 – 95.9)  14.9 ± 12.1  (0 – 72.1) |
| Mentation | Alert  Depressed | 1.9 ± 1.7  (0 – 13.7)  2.1 ± 2.0  (0.3 – 7.37) | 36.8 ± 23.6  (0 – 97.4)  31.1 ± 28.6  (0.5 – 89.4) | 42.5 ± 18.7  (2.4 – 92.3)  49.3 ± 22.7  (8.7 – 87.6) | | 5.5 ± 5.2  (0 – 45.8)  3.0 ± 3.2  (0 – 9.8) | | 0.3 ± 0.8  (0 – 9.1)  0.1 ± 0.3  (0 – 1.2) | 0 ± 0.1  (0 – 0.7)  0 ± 0.1  (0 – 0.3) | 14.8 ± 13.1  (0 – 95.9)  16.4 ± 10.8  (1.9 – 35.3) |
| Posture | Standing  Sternal recumbency | 1.9 ± 1.7  (0 – 13.7)  2.3 ± 1.9  (0.3 – 7.4) | 37.2 ± 23.9  (0 – 97.4)  29.7 ± 22.0  (0.7 – 79.3) | 42.2 ± 18.8  (2.4 – 92.3)  50.2 ± 18.1  (15.8 – 91.6) | | 5.4 ± 5.3  (0 – 45.8)  4.6 ± 3.5  (0 – 12.7) | | 0.3 ± 0.8  (0 – 9.1)  0.2 ± 0.5  (0 – 1.9) | 0 ± 0.1  (0 – 0.7)  0 ± 0  (0 – 0.2) | 14.9 ± 13.3  (0 – 95.9)  15.3 ± 9.3  (3.5 – 36.5) |
| Head tilt | Absent  Present | 1.9 ± 1.7  (0 – 13.7)  1.2 ± .9  (0.3 – 2.9) | 36.8 ± 24.0  (0 – 97.4)  27.5 ± 12.2  (8.4 – 43.7) | 42.5 ±18.9  (2.4 – 92.3)  54.6 ± 12.3  (36.5 – 81.0) | | 5.4 ± 5.2  (0 – 45.8)  3.9 ± 2.2  (1.0 – 7.3) | | 0.3 ± 0.8  (0 – 9.1)  0.2 ± 0.2  (0 – 0.5) | 0 ± 0.1  (0 – 0.7)  0.1 ± 0.2  (0 – 0.4) | 14.9 ± 13.1  (0 – 95.9)  13.7 ± 10.7  (3.1 – 34.5) |
| Ear position | Normal  Unilateral drooped ear  Bilateral droopy ears | 1.9 ± 1.7  (0 – 13.7)  1.8 ± 1.8  (0.4 – 5.9)  1.8 ± 1.8  (0.3 – 5.4) | 36.7 ± 23.4  (0 – 97.4)  47.6 ± 31.9  (7.4 – 82.8)  24.9 ± 27.0  (0.7 – 80.2) | 42.7 ± 18.5  (2.4 – 92.3)  32.0 ± 17.2  (9.3 – 58.1)  54.7 ± 24.7  (16.1 – 91.6) | | 5.3 ± 4.4  (0 – 26.9)  12.8 ± 18.3  (0.7 – 45.8)  3.0 ± 3.3  (0.2 – 9.8) | | 0.3 ± 0.8  (0 – 9.1)  0.3 ± 0.4  (0 – 0.9)  0.2 ± 0.4  (0 – 1.2) | 0 ± 0.1  (0 – 0.7)  0.1 ± 0.2  (0 – 0.4)  0 ± 0  (0 – 0) | 15.0 ± 12.8  (0 – 95.9)  7.2 ± 7.8  (0.9 – 25.3)  17.2 ± 20.3  (3.1 – 74.7) |
| Nasal discharge | Absent  Unilateral seromucous  Unilateral  muco-purulent  Unilateral purulent  Bilateral serous  Bilateral seromucous  Bilateral  muco-purulent  Bilateral purulent | 1.9 ± 1.8  (0 – 13.7)  2.4 ± 2.5  (0.2 – 7.4)  0.5 ± 0.7  (0 – 1.0)  1.8 ± 1.3  (0.5-3.9)  1.5 ± .6  (0.5 – 2.4)  1.7 ± 1.1  (0.6 – 4.4)  1.7 ± 1.6  (0.5 – 5.1)  1.9 ± 1.6  (0.1 – 7.4) | 36.8 ± 24.2  (0.5 – 97.4)  32.0 ± 10.8  (15.5 – 50.7)  16.8 ± 19.7  (2.9 – 30.8)  32.8 ± 18.0  (2.3 – 70.3)  28.7 ± 22.4  (4.9 – 77.6)  41.2 ± 28.3  (2.6 – 82.8)  32.8 ± 25.4  (0 – 60.7)  40.0 ± 22.4  (3.7 – 81.8) | 43.0 ± 19.2 (2.4 – 91.6)  38.6 ± 8.6  (30.8 – 51.8)  35.3 ± 17.3  (23.1 – 47.6)  45.9 ± 16.4  (19.5 – 80.3)  52.8 ± 19.0  (16.1 – 92.3)  38.7 ± 23.3  (9.3 – 79.4)  40.5 ± 16.7  (18.5 – 64.5)  41.3 ± 16.3  (12.6 – 82.4) | | 5.2 ± 4.3  (0 – 24.3)  7.4 ± 8.9  (1.9 – 26.9)  2.6 ± 0.9  (1.9 – 3.2)  6.2 ± 3.0  (2.6 – 12.4)  9.4 ± 11.8  (0 – 38.4)  3.9 ± 3.3  (0.4 – 12.3)  9.8 ± 16.3  (0.7 – 45.8)  4.8 ± 4.2  (0 – 16.8) | | 0.3 ± 0.8  (0 – 9.1)  1.2± .2  (0 – 6.2)  0 ± 0  (0 – 0)  0.3 ± 0.4  (0 – 1.1)  0.1 ± 0.3  (0 – 0.7)  0.4 ± 1.0  (0 – 3.8)  0.2 ± 0.2  (0 – 0.7)  0.3 ± 0.5  (0 – 0.9) | 0 ± 0.1  (0 – 0.7)  0 ± 0  (0 – 0)  0 ± 0  (0 – 0)  0 ± 0.1  (0 – 0.2)  0.1 ± 0.2  (0 – 0.5)  0 ± 0.1  (0 – .2)  0.1 ± 0.1  (0 – 0.7)  0 ± 0.1  (0 – 0.5) | 14.8 ± 12.9  (0 – 95.9)  20.8 ± 12.0  (3.7 – 32.6)  45.3 ± 37.9  (18.5 – 72.1)  14.7 ± 5.9  (7.1 – 23.0)  8.9 ± 5.5  (2.8 – 17.9)  15.7 ± 18.3  (0.5 – 74.7)  16.6 ± 13.3  (0.9 – 43.2)  13.6 ± 10.5  (2.0 – 43.0) |
| Ocular discharge | Absent  Present | 1.9 ± 1.7  (0 – 13.7)  1.7 ± 1.9  (0.2 – 7.7) | 36.8 ± 23.8  (0 – 97.4)  31.6 ± 24.2  (2.6 – 81.9) | | 42.7 ± 18.8  (2.4 – 92.3)  45.9 ± 20.9  (9.7 – 75.2) | | 5.3 ± 5.2  (0 – 45.8)  6.3 ± 5.4  (0.4 – 20.6) | 0.3 ± 0.8  (0 – 9.1)  0.1 ± 0.2  (0 – 0.7) | 0 ± 0.1  (0 – 0.7)  0.1 ± 0.2  (0 – 0.6) | 14.8 ± 12.8  (0 – 95.9)  16.0 ± 18.6  (0 – 74.7) |
| Spontaneous cough | Absent  Present | 1.8 ± 1.7  (0 – 13.7)  2.1 ± 1.9  (0 – 9.7) | 35.8 ± 24.1  (0 – 97.4)  39.4 ± 22.7  (2.3 – 87.4) | | 42.8 ± 19.2  (2.4 – 92.3)  43.1 ± 17.6  (10 – 80.3) | | 5.6 ± 5.5  (0 – 45.8)  4.5 ± 3.5  (0 – 16.8) | 0.3 ± 0.7  (0 – 6.2)  0.3 ± 1.1  (0 – 9.1) | 0 ± 0.1  (0 – 0.7)  0 ± 0.1  (0 – 0.7) | 15.5 ± 13.5  (0 – 95.9)  12.6 ± 10.7  (0 – 61.7) |
| Fever | Absent  Present | 1.9 ± 1.8  (0 – 13.7) | 36.9 ± 23.7  (0 – 97.4) | | 42.6 ± 18.6  (2.4 – 92.3) | | 5.4 ± 5.1  (0 – 45.8) | 0.3 ± 0.9  (0 – 9.1) | 0 ± 0.1  (0 – 0.7) | 14.8 ± 12.8  (0 – 95.9) |
| Laryngeal cough reflex | Negative  Positive | 1.8 ± 1.7  (0 – 13.7)  3.4 ± 2.3  (0.5 – 7.7) | 36.2 ± 23.6  (0 – 97.4)  52.1 ± 26.9  (6.5 – 84.8) | | 43.0 ± 18.9  (2.4 – 92.3)  38.3 ± 18.7  (12.6 – 67.1) | | 5.4 ± 5.2  (0 – 45.8)  3.1 ± 2.3  (0.4 – 7.2) | 0.3 ± 0.8  (0 – 9.1)  0.3 ± 0.4  (0 – 0.9) | 0 ± 0.1  (0 – 0.7)  0 ± 0.1  (0 – 0.3) | 15.1 ± 13.0  (0 – 95.9)  6.3 ± 10.2  (0 – 32.5) |
| Tracheal cough reflex | Negative  Positive | 1.8 ± 1.7  (0 – 13.7)  2.2 ± 1.6  (0.5 – 7.7) | 35.4 ± 23.5  (0 – 97.4)  43.6 ± 24.8  (0.5 – 89.4) | | 43.3 ± 19.0  (2.4 – 92.3)  40.0 ± 18.3  (8.7 – 89.6) | | 5.6 ± 5.3  (0 – 45.8)  4.2 ± 4.0  (0 – 20.6) | 0.3 ± 0.8  (0 – 9.1  0.3 ± 0.7  (0 – 3.2) | 0 ± 0.1  (0 – 0.7)  0 ± 0.1  (0 – 0.7) | 15.4 ± 13.4  (0 – 95.9)  11.8 ± 10.2  (0 – 35.3) |
| Auscultation lung | Normal  Abnormal | 1.8 ± 1.8  (0 – 13.7)  1.9 ± 1.6  (0 – 9.7) | 36.5 ± 23.9  (0 – 87.4)  36.6 ± 23.8  (0.5 – 97.4) | | 41.6 ± 18.3  (2.5 – 92.3  43.9 ± 19.3  (2.4 – 91.6) | | 5.6 ± 6.1  (0 – 45.8)  5.2 ± 4.2  (0 – 24.3) | 0.2 ± 0.6  (0 – 6.2)  0.4 ± 0.9  (0 – 0.7) | 0 ± 0.1  (0 – 0.6)  0 ± 0.1  (0 – 0.7) | 16.1 ± 14.7  (0 – 95.9)  13.9 ± 11.4  (0 – 74.7) |

Values represent mean ± standard deviation (Min.-Max.). TNCC = total nucleated cell count; Neu = neutrophils; Mac = macrophages,
Lym = lymphocytes, Eo = eosinophils; Baso = basophils; Epi = epithelial cells.
